# The complete chloroplast genome sequence of *Ficus hirta* (Moraceae)

**DOI:** 10.1080/23802359.2019.1689867

**Published:** 2019-11-13

**Authors:** Yinrong Liu, Wenna Chen, Fang Li, Chan Li, Xuena Xie, Zhi Chao, Enwei Tian

**Affiliations:** a School of Traditional Chinese Medicine, Southern Medical University, Guangzhou, China;; b College of Landscape and Ecological Engineering, Hebei University of Engineering, Handan, China

**Keywords:** *Ficus hirta*, complete chloroplast genome, phylogenetic analysis

## Abstract

The dry root (Radix Fici Hirtae) of *Ficus hirta* has been used as a traditional herbal medicine in Ling nan regions of China for a long time. As its large market demand, the wild resources of *F. hirta* have sharply reduced. It is necessary to conduct the study of conservation genetics. However, there is still lack of complete genome information for the research on evolutionary biology, population genetics and phylogeography of this species. Here, we sequenced the complete chloroplast (CP) genome of *F. hirta* using Next Generation Sequencing technology (NGS). The CP genome of *F. hirta* is 160,374 bp in length, which contains a large single-copy (LSC) region of 88,446 bp, a small sing-copy (SSC) region of 18,134 bp, and two inverted repeat (IRa and IRb) regions of 26,897 bp. A total of 130 genes were successfully annotated containing 85 protein-coding genes, 37 tRNA genes and 8 rRNA genes. Phylogenetic analysis support genus Ficus is monophyletic and *F. hirta* is closely related to *F. carica* within this genus.

The dry root (Radix Fici Hirtae) of *Ficus hirta* Vahl is a traditional herbal medicine in Ling nan regions of China with a long history for application. Radix Fici Hirtae has the effects of strengthening the spleen, nourishing the lung, removing dampness and relaxing muscles (Ma and Zhang 2010). In addition to medical values, Radix Fici Hirtae is drug-food homologous, whose food ingredient is favored by the Ling nan residents of China (Shi et al. 2013; Wu et al. 2013). With the development of all kinds of products sourced from Radix Fici Hirta, the wild resources of *F. hirta* have reduced increasingly (Dong et al. 2014). It is necessary to conduct management and protection of wild resources of *F. hirta* to prevent the resources depletion. Although, Radix Fici Hirta has been widely researched and applied, they mostly focused on quality standard, chemical composition, pharmacological action etc. (Luo and Jiang 2014), there is still lack of study on resources conservation of *F. hirta*, such as the aspects of evolutionary biology, population genetics, phylogeography etc. Therefore, we sequenced the complete chloroplast genome of *F. hirta* and aimed to obtain much more genetic information of this species.

A sample of *F. hirta* was collected in XinYi city, China (N:22° 18' 09''; E:111°10' 32''), and deposited at the herbarium of School of Traditional Chinese Medicine, Southern Medical University (specimen code: CYR-1). CTAB method was used to extract total genomic DNA (Yang et al. 2014). Illumina paired-end (PE) library was pre-pared and sequenced on an illumina Hiseq4000 platform (Novogene biotechnology Co.Ltd, Bejing, China). The chloroplast genome was assembled using SPAdes version 3.11.1 (Bankevich et al. 2012) with the chloroplast genome of *Ficus carica* as a reference (GenBank accession: KY635880). The chloroplast genome of *F. hirta* was annotated using Geneious version 11.0.4 (Kearse et al. 2012) and Plastid Genome Annotator (PGA) (Qu et al. 2019) and deposited in GenBank (Accession NO.: MN364706).

The complete chloroplast genome of *F. hirta* is 160,374 bp in length, including a large single-copy (LSC) region of 88,446 bp, a small single-copy(SSC) region of 18,134 bp, and two inverted repeat (IRa and IRb) regions of 26,897 bp. The GC content of cp genome of *F. hirta* is 36.0%. The CP genome comprises a total of 130 genes, including 85 protein-coding, 8 ribosomal RNA (rRNA) genes, and 37 transfer RNA (tRNA) genes. Fifteen genes (trnK-UUU, rps16, trnG-UCC, atpF, rpoC1, trnL-UAA, trnV-UAC, petB, petD, rpl16, rpl2, ndhB, trnI-GAU, trnA-UGC and ndhA) contain one intron, two genes (clpP, ycf3) have two introns. A trans-splicing gene was found (rps12 gene).

The phylogenetic reconstruction including the represents of genus Ficus, and closely related genus Antiaris, Broussonetia, Morus (Moraceae) was conducted. 11 chloroplast genome sequences downloaded from GenBank and one (*F. hirta*) were used for phylogenetic analysis (Figure 1). All sequences were aligned with the MAFFT v7.037 (Katoh and Standley 2013). Phylogenetic reconstruction was performed with MEGA v7.0 based on the neighbour-joining (NJ) analysis (Tamura et al. 2011). The results support genus Ficus is monophyletic and *F. hirta* is closely related to *F. carica* within this genus (Figure 1).

**Figure 1.**
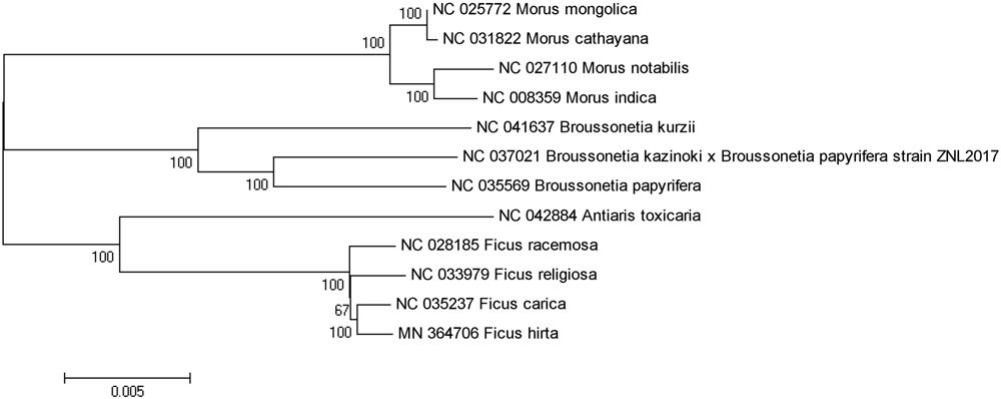
Neighbour-joining (NJ) phylogenetic tree constructed from 12 complete chloroplast genome sequences of Moraceae family based on K2-P distance with 1000 bootstrap replicates. The bootstrap support values are indicated at the nodes.
